# How to Modify (Implicit) Evaluations of Fear-Related Stimuli: Effects of Feature-Specific Attention Allocation

**DOI:** 10.3389/fpsyg.2016.00717

**Published:** 2016-05-13

**Authors:** Jolien Vanaelst, Adriaan Spruyt, Jan De Houwer

**Affiliations:** Department of Psychology, Ghent UniversityGhent, Belgium

**Keywords:** feature-specific attention allocation, selective attention, spider fear, implicit evaluation, extinction

## Abstract

We demonstrate that feature-specific attention allocation influences the way in which repeated exposure modulates implicit and explicit evaluations toward fear-related stimuli. During an exposure procedure, participants were encouraged to assign selective attention either to the evaluative meaning (i.e., Evaluative Condition) or a non-evaluative, semantic feature (i.e., Semantic Condition) of fear-related stimuli. The influence of the exposure procedure was captured by means of a measure of implicit evaluation, explicit evaluative ratings, and a measure of automatic approach/avoidance tendencies. As predicted, the implicit measure of evaluation revealed a reduced expression of evaluations in the Semantic Condition as compared to the Evaluative Condition. Moreover, this effect generalized toward novel objects that were never presented during the exposure procedure. The explicit measure of evaluation mimicked this effect, although it failed to reach conventional levels of statistical significance. No effects were found in terms of automatic approach/avoidance tendencies. Potential implications for the treatment of anxiety disorders are discussed.

## Introduction

Attitudes drive behavior (Allport, 1935) and are therefore often targeted as a leverage point for behavioral change. Importantly, behavior is determined not only by carefully constructed opinions of what we like or dislike but also by spontaneous evaluations that can take place under automaticity conditions (Fazio, 1990; Greenwald and Banaji, 1995). To promote behavioral change, it may thus be beneficial or even necessary to develop intervention strategies that allow for a change of these implicit evaluations. In line with this reasoning, it has been demonstrated that experimentally induced changes in the automatic evaluation of alcohol-related stimuli can result in a corresponding change in alcohol consumption (e.g., Houben et al., 2010). Similar findings have been reported in the domain of implicit self-esteem (e.g., Baccus et al., 2004; Conner and Barrett, 2005; Clerkin and Teachman, 2010), consumer research (e.g., Gibson, 2008) and social cognition (e.g., Rydell and McConnell, 2006).

In the present research, we examined the viability of a novel strategy to reduce implicit evaluations toward fear-related stimuli. This new approach is based on the observation that automatic evaluative stimulus processing is dependent upon feature-specific attention allocation (FSAA), that is, the amount of attention assigned to a specific stimulus feature such as valence, threat-value, gender, size, etc. As an example, consider the evaluative priming studies by Spruyt et al. (2009; for related findings see Spruyt et al., 2012, 2015; Spruyt, 2014; Spruyt and Tibboel, 2015; see also Kiefer and Brendel, 2006; Kiefer and Martens, 2010). Evaluative priming studies typically consist of a series of trials in which participants are asked to respond to a target stimulus (e.g., a picture of a cute baby) that is preceded by a briefly presented prime stimulus (e.g., a picture of a spider). Crucially, the evaluative congruence of the prime-target pairs is manipulated: whereas both stimuli share the same evaluative connotation on some trials (e.g., a positive prime followed by a positive target), other trials consist of incongruent prime-target pairs (e.g., a positive prime followed by a negative target). A typical observation is a performance benefit in speed and/or accuracy for congruent trials relative to incongruent trials. This effect can come about only if participants process the evaluative tone of the primes and can therefore be used as an index of stimulus evaluation. Despite numerous studies attesting to the unconditional, automatic nature of this so-called ‘evaluative priming effect’ (see Klauer and Musch, 2003), Spruyt and colleagues demonstrated that the occurrence of this effect is restricted to conditions that maximize selective attention for the evaluative stimulus dimension. Moreover, adding to the generality of the FSAA framework, a number of recent studies confirmed that FSAA exerts similar effects on various other behavioral (Everaert et al., 2013a) and neuropsychological markers (Everaert et al., 2013b) of implicit evaluation.

Based on the FSAA framework, one can identify two different pathways to reduce implicit evaluations toward fear-related stimuli. First, it may be hypothesized that experimentally induced changes in FSAA at *time 1* can determine the likelihood that one engages in automatic processing of a stimulus feature at *time 2*. More specifically, the FSAA framework naturally predicts that evaluative responses toward fear-related stimuli at *time 2* are less likely to come about in individuals who have learned to refrain from evaluative stimulus processing *at time 1*. Second, the impact of FSAA upon implicit evaluations may be exploited as a means to increase the efficacy of an extinction treatment. Research has repeatedly shown that evaluative responses are highly resistant to extinction (Craske, 1999; De Houwer et al., 2001; Hofmann et al., 2010; Hallion and Ruscio, 2011; Krypotos et al., 2015). This resistance-to-extinction could be due to the fact that, during an extinction treatment, the attitude object automatically evokes an evaluative response that consistently reaffirms the information acquired during the preceding evaluative learning episodes (Martin and Levey, 1978; Lewicki et al., 1992). The FSAA framework predicts, however, that an encounter with an attitude object is less likely to result in an evaluative response if attention is directed away from the evaluative stimulus dimension, thereby allowing for a potential disconfirmation of the preceding evaluative learning episodes (e.g., Lovibond, 2011, see also Sanbonmatsu et al., 2007). Accordingly, one may predict that the extinction rate of evaluative responses toward fear-related stimuli must be contingent upon the degree to which attention is assigned to other, non-evaluative (semantic) stimulus features.

To shed light on these issues, we conducted an exposure study in which FSAA was either directed toward or away from the evaluative stimulus dimension during the exposure phase. Participants were presented with a series of real-life pictures, the content of which varied along two orthogonal semantic dimensions: valence (positive vs. negative) and animacy (living vs. non-living). Participants were asked to categorize all stimuli either as living vs. non-living (i.e., the Semantic Condition) or as positive vs. negative (i.e., the Evaluative Condition). Participants in the Semantic Condition were thus encouraged to assign selective attention to the non-evaluative semantic features of the stimulus materials whereas participants in the Evaluative Condition were encouraged to assign selective attention to the evaluative tone of the stimulus materials. Crucially, the category of negative, living stimuli included pictures of spiders only, thereby allowing for a test of the hypothesis that a manipulation of FSAA can be exploited as a means to reduce evaluations toward fear-related stimuli.

To register the impact of this intervention strategy, we used both a measure of implicit evaluation (i.e., the Affect Misattribution Paradigm; Payne et al., 2005) and explicit evaluative ratings. In addition, because positive and negative evaluations are assumed to promote automatic approach and avoidance behavior, respectively (Solarz, 1960; Krieglmeyer et al., 2010), we also included a Relevant–Stimulus Response Compatibility task aimed at capturing these motivational response tendencies (i.e., the R–SRC task; Mogg et al., 2003). We hypothesized that each of these measures would reveal less negative evaluations toward spiders in the Semantic Condition as compared to the Evaluative Condition. In addition, we included (novel) exemplars that were not presented during the exposure phase to examine the extent to which the impact of our manipulation would generalize to novel (transfer) stimuli.

## Materials and Methods

### Participants

Sixty-one students of Ghent University (13 men, 48 women) participated in the experiment and received 5€ in exchange for their help. In total, two participants in the Evaluative Condition and two participants in the Semantic Condition were excluded from analysis. One participant was excluded due to a technical error. Two other participants were excluded because their error rates in the R–SRC task (i.e., 22.66 and 21.09%) exceeded the outlier criterion of 2.5 standard deviations above the sample mean (*M* = 8.49%, *SD* = 4.69%). Finally, one participant was excluded because her mean reaction time in the R–SRC task (i.e., 992 ms) exceeded the outlier criterion of 2.5 SDs above the grand mean (*M* = 713 ms, *SD* = 100 ms). Unless otherwise mentioned, results were not contingent upon inclusion or exclusion of these participants. The final sample consisted of 11 men and 46 women ranging between 18 and 36 years of age (*M* = 23.39, *SD* = 3.27). Power analyses revealed that, given this sample size, the power to detect a small effect (i.e., Cohen’s *d* = 0.2), a medium-sized effect (i.e., Cohen’s *d* = 0.5), or a large effect (i.e., Cohen’s *d* = 0.8) was 12.46, or 0.84, respectively. The reported research was conducted in accordance with the ethical standards of the institutional ethics committee and with the 1964 Helsinki declaration and its later amendments. All participants gave their informed consent prior to their inclusion in the study.

### Materials

The stimulus materials used for the main dependent measures were eight positive and eight negative color pictures (328 pixels wide and 246 pixels high), 13 of which (i.e., eight positive and five negative) were chosen based on norm data collected by Spruyt et al. (2002). Several of these pictures originated from the International Affective Picture System (i.e., IAPS; Lang et al., 1999). On a scale ranging from -5 (“very negative”) to +5 (“very positive”), the mean valence rating of the negative stimuli was significantly smaller than zero, *M* = -2.08, *SD* = 1.05, *t*(4) = -4.44, *p* < 0.05. The mean valence rating of positive stimuli was significantly larger than zero, *M* = 2.00, *SD* = 0.72, *t*(7) = 7.85, *p* < 0.001. In addition to these IAPS pictures, three pictures of spiders were included. The final sample of 16 stimuli varied on two orthogonal semantic dimensions (i.e., valence and animacy), creating four stimulus categories. The category of living, negative stimuli was represented by four pictures of spiders. Each of the other three categories was represented by a mixture of pictures depicting different themes (see Appendix). For each individual participant, these 16 pictures were split in two semi-random subsets, each consisting of two pictures from each stimulus category. One of these subsets was used during the exposure phase of the experiment (hereafter referred to as *experimental stimuli*). The second set was used to test for transfer effects after the exposure phase (hereafter referred to as *transfer stimuli*).

For the AMP, 200 different Chinese pictographs served as target stimuli. All Chinese pictographs were presented in white and were 256 pixels wide and 256 pixels high. During the R–SCR task, participants were asked to make a (white) manikin move away or toward the stimuli presented in the center of the computer screen (see below). The manikin was about 51 pixels wide and 79 pixels high.

All computer tasks were run on a Dell Optiplex GX520 computer. An Affect 4.0 program (Spruyt et al., 2010) controlled the presentation of the stimuli as well as the registration of the responses. All stimuli were presented against the black background of a 19 inch computer monitor (100 Hz).

For exploratory reasons, we also administered a series of questionnaires. First, the Depression Anxiety Stress Scale (DASS, Lovibond and Lovibond, 1995) was used to measure levels of depression, anxiety and stress in the week preceding the experiment. The DASS consists of 42 statements (e.g., *I found it difficult to relax*) which are to be rated on a four-point Likert Scale ranging from 0 (not at all) to 3 (very much). The internal consistency of the DASS is typically very good, with Cronbach’s alpha’s for the different subscales ranging between 0.83 and 0.91 (de Beurs et al., 2001). In the present sample, Cronbach’s alpha’s were 0.86, 0.89, and 0.88 for the anxiety, stress, and depression subscales, respectively. Second, to capture the extent to which participants tended to experience, on average, a positive or negative mood, they were asked to complete the Positive and Negative Affect Schedules (PANAS, Watson and Clark, 1988). Each mood scale included 10 mood descriptors (e.g., *proud*, *guilty*) and participants were asked to rate each item on a five-point Likert scale ranging from 1 (not at all) to 5 (extremely). The internal consistency of the PANAS is high, both for the English version (i.e., Cronbach’s alpha’s equal or larger than 0.80; Watson and Clark, 1988) and the Dutch version (i.e., Cronbach’s alpha’s equal or larger than 0.79; Engelen et al., 2006). In the present sample, Cronbach’s alpha equaled 0.85 for the positive subscale and 0.78 for the negative subscale. Third, to capture state anxiety, participants completed the Dutch version of the state anxiety subscale of the State Trait Anxiety Inventory (STAI-S, Spielberger et al., 1970; Van der Ploeg et al., 1980). Each item of the STAI-S (e.g., *I feel frightened*) was scored on a four-point Likert scale ranging from 1 (not at all) to 4 (very much). Both the original and the Dutch version of the STAI-S exhibit good internal consistency (i.e., Cronbach’s alpha’s equals or larger than 0.89; Van der Ploeg et al., 1980; Barnes et al., 2002). In the present sample, Cronbach’s alpha was 0.92. Finally, the Fear of Spiders Questionnaire (FSQ, Szymanski and Donohue, 1995) was administered to assess spider fear. The FSQ consists of 18 statements (e.g., *I do anything to avoid a spider*) which are to be rated on an eight-point Likert scale ranging from 0 (completely disagree) to 7 (completely agree). Both Szymanski and Donohue (1995) and Muris and Merkelbach (1996) reported very high internal consistency estimates for the FSQ (i.e., Cronbach’s alpha’s equal or larger than 0.92). Likewise, Cronbach’s alpha in the present sample equaled 0.97.

### Procedure

**FIGURE 1:**
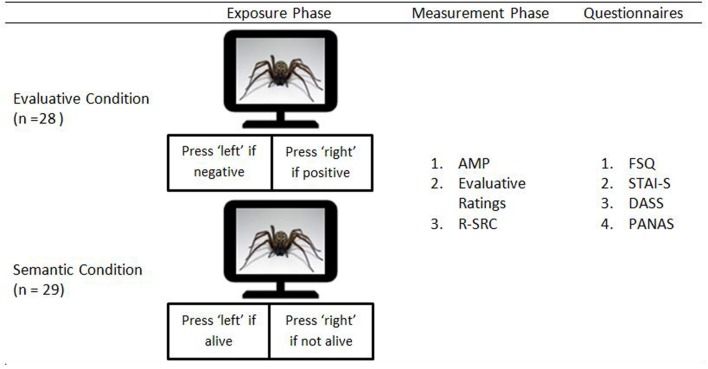
**Schematic overview of the experimental procedure**.

The experiment consisted of an exposure phase followed by an assessment phase (see **Figure 1**). During the exposure phase, the experimental stimuli were each presented 8 times in a random order (i.e., 64 trials). Participants were randomly assigned to either the Evaluative Condition (*n* = 28) or the Semantic Condition (*n* = 29). Participants assigned to the Evaluative Condition were asked to categorize these stimuli on the basis of their evaluative meaning (i.e., positive vs. negative). Participants assigned to the Semantic Condition were asked to categorize these stimuli in terms of the animacy dimension (i.e., living or not living). Selective attention for the evaluative stimulus dimension was thus maximized in the Evaluative Condition and minimized in the Semantic Condition.

Each trial started with the presentation of a fixation cross for 500 ms. Next, after an inter-stimulus interval of 500 ms, a stimulus was presented until a response was registered. Participants in the Evaluative Condition pressed the left key if the stimulus was negative and the right key if the stimulus was positive. Participants in the Semantic Condition pressed the left key if the stimulus depicted an object and the right key if the stimulus depicted a living creature. In case of an erroneous response, a 500-ms error message (i.e., ‘FOUT!’)^1^ appeared. The inter-trial interval varied randomly between 500 and 1500 ms.

During the subsequent measurement phase, participants first completed an AMP, modeled after the recommendations of Payne et al. (2005). Both the experimental stimuli and the transfer stimuli were used as primes and were presented once in an intermixed, random order (i.e., 16 trials in total). It may be noted that we deliberately chose to implement a small number of AMP trials as the AMP requires participants to evaluate stimuli. Using a higher number of trials might thus have interfered with the attention manipulation. Each trial started with the presentation of a fixation cross for 500 ms, followed by an inter-stimulus interval of 500 ms and a 75-ms presentation of a prime stimulus. Next, 125 ms after the offset of the prime stimulus, a randomly selected Chinese pictograph was presented for 100 ms. Finally, immediately following the presentation of the Chinese pictograph, a black-and-white masking stimulus was presented until a response was registered. Participants were instructed to press the left key if they considered the Chinese pictograph to be less pleasant than the average Chinese pictograph and the right key if they considered the Chinese pictograph to be more pleasant than average. The inter-trial interval varied randomly between 500 and 1500 ms.

Following the AMP, participants were asked to rate the evaluative meaning of the experimental and the transfer stimuli using a rating scale ranging from -100 to +100. Each stimulus was presented until a response was triggered and the trial list was completely random. The inter-trial interval varied randomly between 500 and 1500 ms.

Next, participants completed the R–SRC-task, modeled after Spruyt et al. (2013). On each trial, either an experimental stimulus or a transfer stimulus was presented in the middle of the computer screen. Simultaneously, a manikin was presented either below or above the position of the stimulus (i.e., counterbalanced across trials and individual stimuli). During a first block of trials, participants were asked to move the manikin away from positive stimuli and toward negative stimuli (i.e., incongruent trials) using the arrow keys of a standard computer keyboard. In a second block of trials, participants were asked to move the manikin away from negative stimuli and toward positive stimuli (i.e., congruent trials). They were allowed to move the manikin in any direction, but a loud beeping sound was delivered if the initial movement of the manikin was incorrect. A trial ended if the manikin reached either its highest or its lowest possible position in the accurate direction (i.e., the upper/lower edge of the computer or picture, 10 steps in each direction). Each stimulus was presented exactly twice during each block, leading to a total of 64 trials. The inter-trial interval varied randomly between 500 and 1500 ms.

Finally, at the end of the experiment, participants were asked to complete the FSQ, STAI-S, DASS, and PANAS (fixed order).

## Results

Preliminary analyses revealed that none of the critical effects was qualified by an interaction with stimulus type (i.e., experimental versus transfer stimuli) or animacy (i.e., living versus non-living stimuli), all *F’*s < 2.70. Accordingly, the data were collapsed across these variables. Note, however, that summary statistics for each cell of the design are provided in **Table 1**.

**Table 1:** Mean scores for dependent measures as a function of Condition (SD’s in parentheses).

Condition	Stimulus type				
		Experimental stimuli		Transfer stimuli	
	All stimuli	Living creatures	Objects	Living creatures	Objects
**AMP scores**
Semantic	0.07 (0.36)ˆ**	-0.03 (0.63)ˆ*	0.10 (0.52)	0.12 (0.55)	0.10 (0.54)ˆ**
Evaluative	0.42 (0.47)ˆ**	0.41 (0.65)ˆ*	0.38 (0.66)	0.38 (0.55)	0.52 (0.55)ˆ**
**Evaluative ratings**
Semantic	118.38 (30.55)	126.78 (54.53)	106.47 (30.21)	128.34 (40.24)	111.91 (37.87)
Evaluative	131.29 (30.77)	137.55 (41.29)	121.34 (39.18)	138.36 (37.78)	127.89 (36.73)
**R–SRC Scores (D600)**
Semantic	0.46 (0.44)	0.71 (0.67)	0.44 (0.68)	0.47 (0.54)	0.42 (0.56)
Evaluative	0.49 (0.36)	0.42 (0.59)	0.62 (0.67)	0.35 (0.53)	0.67 (0.72)

p < 0.10, *p < 0.05, **p < 0.01.

AMP scores were calculated by subtracting the proportion of pleasant judgments on trials depicting negative stimuli from the proportion of pleasant judgments on trials depicting positive stimuli. Likewise, evaluative rating scores were calculated by subtracting the mean rating of negative stimuli from the mean rating of positive stimuli. For the R–SRC task, individual scores were obtained using the so-called D600 algorithm (Greenwald et al., 2003). First, all reaction times slower than 300 ms and higher than 10,000 ms were removed (0.16%). Second, for each block of trials, reaction times observed on error trials (8.47%) were replaced by the mean of correct latencies plus a 600-ms penalty. Third, a pooled *SD* was calculated based on correct trials and corrected error trials. Fourth, the mean response latency observed on congruent trials was subtracted from the mean response latency observed on incongruent trials. Finally, this difference score was divided by the pooled *SD*. For all dependent measures, higher values correspond with a more marked difference between positive and negative stimuli.

A bootstrapping approach was adopted to examine the reliability of the AMP effect and the R–SRC effect. For each measure and for each of 10,000 runs, the data of each individual participant were split in two equally sized, random sets. Two AMP scores and two R–SRC scores were then calculated, one for each subset. Next, for each measure and for each individual run, the correlation between the two scores was computed and Spearman–Brown corrected. The split-half reliability coefficient was then obtained by computing the average of these 10,000 correlations. For the AMP effect, the split-half reliability coefficient equaled 0.76. The split-half reliability coefficient for the R–SRC effect was 0.71.

Overall, each of the three dependent measures revealed a more favorable evaluation of positive stimuli as compared to negative stimuli, *t*(56) = 4.08, *p* < 0.001, *d* = 0.54, *t*(56) = 30.30, *p* < 0.001, *d* = 4.01, *t*(56) = 9.02, *p* < 0.001, *d* = 1.19, for the AMP, the evaluative ratings and the R–SRC, respectively. More importantly, a one-way ANOVA with Condition as a between subjects factor revealed that the AMP scores were reliably different in both conditions, *F*(1,55) = 9.74, *p* < 0.01, η^2^ = 0.15. Follow-up analysis revealed a significant AMP effect in the Evaluative Condition, *t*(27) = 4.70, *p* < 0.001, *d* = 0.89, but not in the Semantic Condition, *t*(28) = 1.10, *p* = 0.28, *d* = 0.20. Numerically, the rating data mimic these results, but the main effect of Condition failed to reach statistical significance, *F*(1,55) = 2.53, *p* = 0.12, η^2^ = 0.04^2^. In contrast, the R–SRC scores did not reveal a reliable difference between the two conditions, *F* < 1 (see **Table 1**).

For each of the dependent measures (i.e., evaluative ratings, AMP scores and R–SRC scores), we also examined whether the effect of the attention manipulation was moderated by inter-individual differences as measured by the questionnaires. While there was no evidence for such a moderation for the PANAS, the DASS, and the STAI-S, all *F’*s < 1, an ANCOVA did reveal a significant interaction between the Condition factor and the FSQ score, at least for the AMP data, *F*(1,53) = 4.72, *p* < 0.05, η^2^ = 0.08. Reassuringly, more extreme levels of spider fear were associated with a more pronounced difference in AMP Scores between the Evaluative and the Semantic Condition. A similar effect did not emerge for the evaluative ratings and the R–SRC data.

Finally, correlational analyses revealed a significant correlation (*r* = 0.36) between the AMP scores and the evaluative ratings, *t*(55) = 2.86, *p* < 0.01. In contrast, neither the AMP scores nor the evaluative ratings correlated with the R–SRC scores, *t*s < 1. Interestingly, the correlation between the FSQ scores and the AMP scores was substantial in the Evaluative Condition, *r* = 0.47, *t*(26) = 2.70, *p* < 0.05. More extreme levels of spiders fear were associated with more extreme AMP Scores. The correlation between the AMP scores and the FSQ scores did not reach significance in the Semantic Condition, *r* = 0.06, *t* < 1.

## Discussion

The aim of the present research was to test the viability of a new method to reduce the negativity of the (implicit) evaluation of fear-related stimuli. Based on the FSAA-framework developed by Spruyt et al. (2007, 2009; Everaert et al., 2013a), it was hypothesized that the requirement to engage in a non-evaluative processing style during an exposure procedure would impact measures of evaluation during a subsequent measurement phase, for two reasons. First, the attentional focus on non-evaluative stimulus information may carry over from the exposure phase to the test phase, thereby reducing the likelihood and/or intensity of the evaluative response toward fear-related stimuli. Second, participants may be more likely to experience corrective emotional information during an exposure procedure if the likelihood and/or intensity of an evaluative response is minimized during the exposure phase.

To obtain a proof-of-principle for these ideas, we conducted an exposure study in which participants were asked to categorize fear-related pictures (e.g., pictures depicting spiders) either in terms of their evaluative meaning (i.e., Evaluative Condition) or in terms of the animacy dimension (i.e., Semantic Condition). Participants were thus encouraged to assign selective attention to the evaluative and non-evaluative semantic features of stimuli, respectively. In line with our predictions, we observed that implicit evaluations as measured by the AMP were less pronounced in the Semantic Condition as compared to the Evaluative Condition, both for stimuli used during the manipulation phase and novel transfer stimuli. A similar result was obtained with the explicit evaluative ratings, although it must be noted that this effect was statistically not unequivocal (*p* = 0.12, but see Footnote 1). Given that the AMP and the explicit evaluative ratings were substantially correlated, however, we are inclined to attribute the absence of a reliable effect in the explicit valence ratings to a Type-II error.

Interestingly, we also observed that the correlation between the AMP scores and the FSQ scores (i.e., an explicit measure of spider fear) was dependent upon our experimental manipulation. Whereas a strong correlation was found in the Evaluative Condition (*r* = 0.47), there was no evidence for such a relationship in the Semantic Condition (*r* = 0.06). This finding strengthens our claim that FSAA can modulate the automatic evaluation of fear-related stimuli as it suggests that individual differences in automatic evaluation were picked up reliability by the AMP in the Evaluative Condition but not in the Semantic Condition.

The current findings are important because they shed new light on the mixed results that have been reported in the field of Attention Bias Modification (i.e., ABM; MacLeod et al., 2002; Hertel and Mathews, 2011; Beard et al., 2012). In a typical ABM procedure, participants are encouraged to divert their spatial attention away from fear-related stimuli using an adapted version of the dot-probe task (MacLeod et al., 1986). Participants are presented with two briefly presented stimuli (i.e., cues), one of which is a fear-relevant stimulus whereas the other is emotionally neutral. On the majority of the trials, the emotionally neutral stimulus is replaced by a visual probe that requires a response. On the remaining trials, the probe is preceded by the fear-related stimulus. It is expected that the predictive relationship between the nature of the cue and the probe causes participants to selectively direct their spatial attention away from threatening stimuli (Bar-Haim et al., 2007), thereby promoting therapeutic change. Whereas several studies attesting to the therapeutic value of ABM training have appeared in the literature (e.g., Amir et al., 2008), some authors reported that they were unable to obtain supporting evidence for the idea that ABM training can reduce attention bias and subsequent vulnerability to psychological stressors (e.g., Julian et al., 2013). Recent meta-analytical studies also raised concern about the therapeutic efficacy of ABM training (Hallion and Ruscio, 2011; Mogoaşe et al., 2014; Heeren et al., 2015).

Importantly, this mixed pattern of results is readily accounted for on the basis of the FSAA framework. According to this framework, different stimulus dimensions attract attention as a function of current goals and task demands. In a traditional ABM training, attending to the threat value of the cues is beneficial for the task at hand as soon as the difference between threatening and neutral cues is predictive for the location of the target probes. As a result, somewhat ironically, one can expect participants to assign selective attention to the difference between threatening and neutral stimuli as soon as they pick up a contingency between the threat value of the cues and the location of the targets. The observation that successful attempts to change attention bias were not always accompanied by corresponding changes in symptoms (Browning et al., 2010) or even increased reported symptomatology (Baert et al., 2010) is consistent with this viewpoint.

The logic developed here differs from the ABM approach in the sense that participants are (a) encouraged to assign spatial attention to fear-relevant stimuli while (b) prioritizing non-evaluative (semantic) stimulus processing over evaluative stimulus processing. Likewise, there is a marked difference between the current approach and the Emotional Processing Theory (i.e., EPT) of exposure therapy (Foa and Kozak, 1986). According to EPT, fear is represented in a fear structure that can be modified only if it is activated. Accordingly, therapeutic sessions often comprise a controlled confrontation with the fear-evoking stimulus, either *in vivo* or *in vitro*. Therapeutic change is then expected to occur only if and to the extent that participants can integrate corrective information during such an experience. In line with such an approach, the intervention developed here requires participants to focus spatial attention on a threat-evoking stimulus. Nevertheless, our approach is novel in the sense that participants were encouraged to selectively process non-evaluative instead of evaluative stimulus features. As demonstrated by the present findings, this approach may provide an additional means to combat pathological fear, but we hasten to confirm that more research would be needed to firmly substantiate this claims.

Further research would also be needed to deal with two limitations of our study. First, it is insufficiently clear why exactly the R–SRC task failed to pick up a difference between the Evaluative Condition and the Semantic Condition. Importantly, given that the overall R–SRC effect did reach significance in both conditions (i.e., performance was consistently better in the compatible block as compared to the incompatible block), we can safely rule out the possibility that the specific version of the R–SRC task used in this study was simply unsuited to detect automatic approach/avoidance tendencies. It also seems unlikely that a (successful) manipulation of FSAA would selectively affect the (implicit) evaluation of a stimulus but not the degree to which this stimulus triggers automatic approach/avoidance tendencies. After all, the (automatic) evaluation of a stimulus can be defined as a necessary precursor of the (automatic) tendency to approach or to avoidance that stimulus (Deutsch and Strack, 2006; Gawronski and Bodenhausen, 2006). Therefore, as an alternative explanation, we suspect that the temporal order of the implicit measures may have been critical. The AMP was always performed first, followed by the evaluative rating task and the SRC task, respectively. We deliberately opted for a fixed order of assessment tasks because (a) we were primarily interested in the influence of FSAA on implicit evaluations (i.e., the AMP) and (b) we wanted to avoid carry-over effects from other tasks while participants completed the AMP. However, because all three dependent measures required participants to evaluate stimuli, one might argue that each of these tasks may have counteracted the effects of the experimental manipulation to some degree. As a logical consequence, it could be argued that the effects of our exposure procedure were abolished by the time participants completed the R–SRC-task. It would thus be interesting to replicate the present experiment while counterbalancing the order of the measurement tasks. Alternatively, it could be worthwhile to use an adaptation of the R–SRC that is semantically neutral. For example, participants might be asked to respond on the basis of the picture format of the target stimuli (e.g., portrait vs. landscape, see Reinecke et al., 2010).

As a second limitation of our study, one may argue we restricted our sample to non-clinical, unselected participants. It thus remains an open question whether the current findings would replicate in a clinical sample. It may be noted, however, that the effect of FSAA was slightly larger, not smaller, when the analyses were restricted to data stemming from participants with elevated levels of (self-reported) fear of spiders (i.e., FSQ Scores >55, see Huijding and de Jong (2006), *F*(1,19) = 15.41, *p* < 0.001, η^2^ = 0.45. More research will be necessary, however, to document the clinical validity of the current findings as well as the life-time of the extinction effect observed in the present study.

These limitations notwithstanding, our findings support the idea that (implicit) evaluations become less intense if participants are encouraged to assign attention to (non-evaluative) semantic stimulus information. This effect was found for generic evaluative stimuli and fear-related stimuli alike and transferred to non-trained exemplars. More research is needed, however, to establish the generality of this effect, its boundary conditions, and underlying mechanisms.

## Author Contributions

JV significant contributions to the conception of the design, acquisition and analyzing of data, interpretation of data, drafting of manuscript. AS significant contribution to the conception of design, revision of data analysis, critical revision of the manuscript, interpretation of data. JH significant contribution to the conception of design, critical revision of the manuscript, interpretation of data.

## Conflict of Interest Statement

The authors declare that the research was conducted in the absence of any commercial or financial relationships that could be construed as a potential conflict of interest.

## Footnotes

1 The Dutch word ‘FOUT’ translates to the English word ‘WRONG.’

2 It may be noted that the rating data did reveal a significant main effect of Condition if outlying data points were *not* excluded from the analyses, *F*(1,58) = 4.37, *p* < 0.05, η^2^ = 0.07. As anticipated, the difference in mean valence ratings between positive and negative stimuli was larger in the Evaluative Condition (*M* = 131.93) compared to the Semantic Condition (*M* = 113.69).
