# Supplementary material for: How to Modify (Implicit) Evaluations of Fear-Related Stimuli: Effects of Feature-Specific Attention Allocation
Source: Front Psychol. 2016 May 13;7:717. doi: 10.3389/fpsyg.2016.00717 (PMC4865498; doi:10.3389/fpsyg.2016.00717)
Supplement: Supplementary file 1 [file Data_Sheet_1.DOCX]

**Appendix**

*Description of stimulus materials used for the main dependent measures*

*Pictures depicting negative objects*: car, gun, garbage, fire

*Pictures depicting negative beings*: spider, spider, spider, spider

*Pictures depicting positive objects*: balloons, lollipop, present, air balloon

*Pictures depicting negative beings:* dolphin, squirrel, baby, kitten
